# New Orleans Medical News and Hospital Gazette

**Published:** 1860-05

**Authors:** 


New Orleans Medical News and Hospital Gazette.—The April number of this journal, edited by Drs. Brickell and Fenner, Professors in the New Orleans School of Medicine, seem to be very wrathy respecting the short article penned by the senior editor of the Review for March, 1860, and entitled "Southern Medical Students and Southern Medical Schools." They declare, not in so many words, it is true, but by implication, that they are ready to die in defense of their noble institution, the establishment of which has cost them so much labor, and anxiety, and treasure, and that they will permit no one to cast upon her any imputation, even of the most indirect character. All this is very right, and just, and valiant. Keep your school pure and chaste before the public and the medical profession. Let her be, like Cæsar's wife, above suspicion. To all such sentiments we cordially subscribe. But why, gentlemen, are you abusing the Review, and calling it by hard names? In the article to which you have taken such exception, the great sin which we committed, in your immaculate eyes, was that we did not include you in the same category as the University of Louisiana, in her disclaimer, made through our colleague, Prof. Richardson, respecting her supposed interference in the late stampede in this city. It is here that the shoe pinches; "*hinc illæ lachrymæ.*" We should have been rejoiced if we could have extended to your school a similar courtesy. But we could not; for, although we are not able to assert, from any official documents in our possession, that you sent any telegraphic communications to the medical students of this city, "tendering sympathy and a cordial welcome to your school," yet we were perfectly sure that you had sent such a dispatch to New York, and we had, therefore, a right to assume that you would have been very glad to perform a similar service for the Philadelphia students if they had addressed you upon the subject, which, however, judging from your silence, we conclude they never did. Be this as it may, our ethics are, that when a man does a wrong, he is equally guilty whether he commits the wrong in one place or in another, upon one person or upon another. The fact that the Faculty of the New Orleans School of Medicine sent a dispatch to New York induced us to exclude her from the benefit of the exculpation which we so gladly extended to the University of Louisiana. Personally, we have no preference for either institution; we have kind friends in both, and we wish them both well; but we could not, we must confess, place them upon the same footing in a matter in which one was observing a dignified silence, and the other catering to public excitement.

In order that our readers may fully understand the position of the New Orleans School of Medicine in regard to the late stampede, we extract the following passages from the April number of the *New Orleans Medical News and Hospital Gazette*:—

"1st, then: The only connection the New Orleans School of Medicine ever has had with the seceding students at the North was in her receiving a dispatch from New York City, signed by the 'Alabama Committee,' saying that the Southern students *contemplated* leaving New York, that they had paid for their tickets, and desiring to know on what terms she would receive them; to which dispatch the Faculty sent the following reply:—

"'The Faculty of the New Orleans School of Medicine deeply regret that you have found yourselves under the necessity of leaving the school to which you are attached. They will receive you on condition that you pay for matriculation and graduation fees.'

"2d. The Faculty of the New Orleans School of Medicine have *never* had, *at any time*, *any* communication, of *any* kind whatsoever, with *any* student of medicine, attached to or seceded from *any* school in Philadelphia."

After giving the preambles and resolutions adopted at a meeting of the New Orleans School of Medicine and of the University of Louisiana, the editors of the *New Orleans Medical Gazette* ask the following questions:—

"Has the *Review* been informed by its correspondent whether *either* of the Faculties here ever 'opposed' these proceedings, either publicly or privately?

"Is the *Review* silly enough to believe that the classes of the schools here or elsewhere, could thus freely throw open the doors of the institutions without the positive approbation of the respective Faculties?"

From the last paragraph it is evident that the editors of the *New Orleans Medical Gazette*, who are also, it will be borne in mind, professors in the New Orleans School, directly indorse the act of their pupils, commending them for it, as well as their own Faculty act. We will not be so uncharitable as to suppose that they instigated this act on the part of their students, and yet such a conclusion is with difficulty avoided. The idea that a school is *necessarily* responsible for the acts of its classes, as expressed at public meetings, is simply absurd.

The editors are disposed to taunt us for our imperfect account of the Philadelphia stampede. All history is, to some extent, a fiction, and in attempting to write that of the stampede, we only stated what, at the time, we supposed to be true, and what has not since been successfully contradicted in any of its material points.

